# *In-vitro* and *In-vivo* Antileishmanial Activity of a Compound Derived of Platinum, Oxaliplatin, against *Leishmania Major*

**DOI:** 10.22037/ijpr.2019.15364.13046

**Published:** 2019

**Authors:** Ezatollah Ghasemi, Fatemeh Ghaffarifar, Abdolhossein Dalimi, Javid Sadraei

**Affiliations:** *Department of Parasitology and Entomology, Faculty of Medical Sciences, Tarbiat Modares University, Tehran, Iran.*

**Keywords:** Leishmania, Oxaliplatin, Promastigote, Macrophage, BALB/c mice

## Abstract

This study aimed to evaluate the antileishmanial efficacy of oxaliplatin against *Leishmania major* *(L. major)* both *in-vitro* and *in-vivo*. The IC_50_, CC_50_, and SI of oxaliplatin against promastigotes, murine macrophages, Raw 264.7 cells, and intramacrophage amastigotes of *L. major* were investigated *in-vitro*. The effects of this drug on intracellular amastigotes were also assayed, and the percentage of infectivity and IIR were calculated. Flow cytometry was performed to assay apoptosis, using 50 and 100 µg/mL of oxaliplatin in the promastigotes and macrophages. *In-vivo*, the BALB/c mice were classified into three groups, receiving oxaliplatin, glucantime, and phosphate-buffered saline for one month, respectively. The lesion size, IFN-γ, and IL-4 levels, and parasite burden were also evaluated in the animals. After 72 h, the IC_50_ and CC_50_ of oxaliplatin against promastigotes and macrophages were respectively 0.5 and 66.78 µg/mL. The apoptosis of promastigotes and macrophages using 50 µg/mL of oxaliplatin was 7.25% and 2.14%, respectively, while apoptosis induced at 100 µg/mL was 15.48% and 2.80%, respectively. Similar to the glucantime group, the mice treated with oxaliplatin showed a lower parasite burden and smaller lesions, compared with the PBS group (*p < *0.01). Furthermore, higher IFN-γ levels were reported in mice receiving oxaliplatin in comparison with those receiving PBS (*p < *0.01). The current findings indicated the efficacy of oxaliplatin against promastigote and amastigote forms of *Leishmania* and *L. major-*induced leishmaniasis.

## Introduction

Leishmaniasis is recognized as an important but neglected parasitic disease, involving intracellular protozoa from the genus *Leishmania. *This disease, which is endemic to 98 countries, mostly affects underprivileged classes in both developed and developing countries. Statistics indicate a prevalence of 12 million cases worldwide, and 350 million people are prone to leishmaniasis (1-3). Annually, 1-1.5 million new cases of cutaneous leishmaniasis (CL) and 500 000 new cases of visceral leishmaniasis (VL) are reported (4-6). CL in humans has been attributed to more than 15 species of parasites. It involves different clinical types, including self-limiting but scarring skin lesions and even chronic disorders, such as diffuse cutaneous leishmaniasis (DCL) and mucocutaneous leishmaniasis (7-9).

Leishmaniasis threatens many people worldwide due to the absence of suitable vaccine and treatment, global warming, war, and migration (10-12). Currently, leishmaniasis is controlled by chemotherapeutic compounds (13-15). For more than 70 years, pentavalent antimonials, including meglumine antimonate (glucantime) and sodium stibogluconate, have been the first choice for leishmaniasis treatment. However, high concentrations of drugs, recently required for treatment due to the increased drug resistance of *Leishmania* parasites, have led to their accumulation in tissues (*e.g.*, kidney, heart, and liver) and may even cause serious adverse effects, such as fatal arrhythmias (16, 17).

Amphotericin B (AmpB) and its lipid formulations (*e.g.*, paromomycin and pentamidine) are also used in the event of resistance, intolerance, or contraindication to antimonial compounds (18-20). Overall, application of these anti-leishmanial drugs is limited because of their drawbacks, which include toxicity, high cost, long-term treatment, drug resistance, and invasive administration route (13, 21-24).

In order to overcome the side effects caused by antileishmanial drugs, recent researches have focused on new compounds against leishmaniasis. The similarity in signaling pathways between cancer and parasites has encouraged parasitologists to use anticancer compounds to design new antiparasitic drugs (25). For example, various antineoplastic agents, alone or in combination with other drugs, including artemisinin (26-28), imatinib (29), 5-fluorouracil (30), and tamoxifen (31), have shown anti-parasitic effects. Also, the strong leishmanicidal effect of miltefosine, an anti-cancer drug, has been confirmed; however, its application is limited because of severe gastrointestinal side effects and teratogenic effects during pregnancy (32, 33). 

Anti-neoplastic platinum compounds (*e.g.*, cisplatin, carboplatin, and oxaliplatin) inhibit DNA transcription and replication, leading to cell death and apoptosis induction in actively dividing cells (34). 

In recent research, some platinum compounds, such as carboplatin and cisplatin, have induced leishmanicidal effects in BALB/c mice against *Leishmania donovani* *(L. donovani)* infection, characterized by a decline in parasite burden and an increase in DTH response and multiple biochemical indices (35).

Oxaliplatin (Eloxatin) is recognized as a new antineoplastic platinum derivative with a DACH carrier ligand (34). Therefore, this study aimed to examine the antileishmanial activities of oxaliplatin against *L.* *major* both *in-vitro* and *in-vivo*. To the best of our knowledge, this is the first report of oxaliplatin effects on CL.

## Experimental

*Cultivation of L. major parasites*

The promastigotes (MRHO/IR/75/ER) were cultured in RPMI-1640 medium (Gibco-BRL, USA) and L-glutamine (20 mM), containing 10% fetal bovine serum (FBS; Gibco-BRL, USA), 100 μg/mL of streptomycin (Sigma), and 100 U/mL of penicillin; incubation was then carried out at 26 °C. Using an inverted microscope, the growth of* L. major *promastigotes was examined each day (36).

*Culture of Raw 264.7 macrophage cells*

To evaluate cytotoxicity and obtain an amastigote macrophage culture, the RPMI-1640 medium, containing streptomycin (100 μg/mL), 10% FBS, and penicillin (100 U/mL), was used for culturing at 37 °C (5% CO_2_). The macrophage cell growth was evaluated daily using an inverted microscope. When the cells reached about 80-90% confluence, they were subcultured into a new flask containing fresh cell culture medium.

*Drug preparation*

Oxaliplatin was purchased from Sigma Aldrich Co. (USA) as white powder with 99% purity and a molecular weight of 397.29 g/mol. After dissolving oxaliplatin in distilled water (1 mL) to obtain a 5-mg/mL stock concentration from the drug, it was aliquoted in 0.5 mL vials (Eppendorf tubes) for storage at 4 °C until use. For *in-vitro* experiments, different concentrations of drugs (*i.e.*, 400, 200, 100, 50, 25, 12.5, 6.25, 3.12, 1.56, and 0.78 µg/mL) were prepared using the RPMI-1640 medium.

*Anti-promastigote activity assay, proliferation assay*

To examine the anti-leishmanial effects, *L. major* promastigotes in the logarithmic growth phase (2 × 10^6^ parasites/mL) were cultured in 96-well microtiter plates containing RPMI-1640 medium with 10% FBS; they were then incubated at 24 ± 2 °C overnight. Following incubation, 100 µL of different oxaliplatin and AmpB concentrations were used to treat the promastigotes for 72 h. The promastigotes cultured with no drugs and RPMI-1640 medium with no promastigotes or drugs were respectively used as the negative control and blank; the experiments were repeated three times. The number of promastigotes was counted under an optical microscope to determine the anti-leishmanial effects, using a hemocytometer chamber. In addition, IC_50 _was assayed by non-linear regression analysis. 

*Promastigote viability test*

The *L. major* promastigotes were evaluated in terms of mitochondrial oxidative activity by measuring parasite viability with the colorimetric MTT method (Sigma Aldrich, USA). Briefly, *L. major* promastigotes (2 × 10^6^ cells/mL) were cultured in plates (Nunclon®, Denmark) containing oxaliplatin (400 to 0.78 µg/mL) at 24 °C for 72 h. On the other hand, the positive control was AmpB, and the negative control included promastigotes cultured in RPMI-1640 medium with 10% FBS without the drug. 

Incubation was performed for three hours at 37 °C after adding 5 mg/mL of MTT solution (20 µL) to the wells. Then, DMSO (100 µL/well) was used to solubilize formazan crystals at room temperature for half an hour (37). An ELISA reader (ELX800) was used to read absorbance at 570 nm. In addition, the IC_50 _of *Leishmania* growth was measured by applying non-linear regression analysis (38). The following formula was measured to determine the viability percentage of promastigotes (39).

[(AT–AB)∕(AC–AB)] × 100%

where AB, AC, and AT represent the OD of the blank well, negative control, and treated cells, respectively.

*Cytotoxicity and selectivity index*

Macrophage viability was determined using the MTT method (Sigma Aldrich, USA). Briefly, after seeding murine macrophage Raw 264.7 cells in 96-well microtiter plates in triplicate, incubation was performed overnight at 37 °C. Then, a fresh medium (100 µL) was used to replace the medium. The cells were treated using different oxaliplatin concentrations as serial dilutions, and AmpB was used as the positive control at 37 °C. After incubation for 72 h, 5 mg/mL of MTT solution (20 µL) was poured in the wells, and incubation was repeated at 37 °C for three h. 

The formazan crystals were solubilized in DMSO (100 µL/well) for 30 min at room temperature. The colorimetric reaction was read using an ELISA reader (ELX800) at 570 nm. The non-linear regression analysis was used to measure the CC_50_ value in GraphPad Prism 6.07 (GraphPad Inc., USA). The selective index (SI) was also calculated by measuring the CC_50_ to IC_50_ ratio (38).

$\mathrm{Cell viability}\left(\%\right)=\frac{\left(\mathrm{average absorbance in triplicate drug wells}-\mathrm{average blank wells}\right)}{\mathrm{average absorbabce control wells}}\times 100$

*Anti-amastigote activity*

After seeding murine macrophage-derived Raw 264.7 cells on round glass coverslips in 12-well plates containing RPMI-1640 medium (with 10% FBS), they were incubated at 37 °C overnight. The adherent macrophages were exposed to* L. major* promastigotes (macrophage-parasite ratio1:10) after washing the non-adherent cells with phosphate buffered saline (PBS); they were then placed at 37 °C for 24 h for phagocytosis of promastigotes. 

For the removal of free promastigotes, the infected macrophages were washed in PBS. For treatment, 6.25, 12.5, and 25 µg/mL of oxaliplatin and AmpB as the positive control were used at 37 °C. The medium with the infected macrophages but without the drug was used as the negative control. After 72 h, the coverslips were fixed in methanol and stained with Giemsa stain solution. The percentage of infectivity was assessed for each sample by measuring the ratio of infected macrophages to 300 macrophages in triplicate. The infection index rate (IIR) represents the mean amastigote count per macrophage cell (40, 41).

*Detection of phosphatidyl serin exposure*

An Annexin-V FLUOS staining kit (Bio-vision, USA) was used to evaluate oxaliplatin induced apoptosis. In brief, *L. major* promastigotes in murine macrophage derived Raw 264.7 cells were exposed to 50 and 100 µg/mL concentrations of oxaliplatin, as mentioned above. Promastigotes and macrophages exposed to no drugs were used as the control groups. After 72 h, the cells were washed in cold PBS and harvested by centrifuging at 14,000 g for 10 min. 

The supernatants were removed and the pellets were resuspended in 500 μL binding buffer, 5 μL annexin-V, and 5 μL propidium iodide (PI). Then, the samples were incubated in darkness at 26 °C for 15 min. Apoptosis was assayed by detecting the PI fluorescence intensity using a FACS Calibur flow cytometer (Becton Diekinson). The results were analyzed using CellQuest software. 

*Experimental animals*

In the present study, Razi Institute of Iran provided female BALB/c mice (4-6 weeks old; 18-20 g). They were kept under optimal conditions (25-26 °C; relative humidity, 55-65%) at the animal facilities of Tarbiat Modares University, Tehran, Iran. The ethics committee of the university approved all animal procedures. We used 36 mice which were infected subcutaneously in the tail base using stationary phase promastigotes. When the nodules were developed in the injection site, the animals were randomly divided into three groups (12 mice per group). Then, animals of each group were divided into three subgroups (n = 4 mice) to measure lesion size, parasite burden, and cytokine assay.

*Development of leishmanial lesions and treatment*

In this study, the mice were infected subcutaneously in the base of the tail using 100 µL of a stationary phase promastigote suspension (1 × 10^6^ parasites). The animals were divided into groups when the nodules developed in the injection site (15-20 days after infection). Group I received 25 mg/kg of oxaliplatin daily for 30 days (i.p. route), group II received 20 mg/kg of glucantime daily for 30 days (IM route), and group III (control) received 200 µL of PBS (i.p. route). From each group, four mice were selected to measure the lesion size. To examine the drug efficacy, the width and length of the lesion were weekly measured using a digital caliper. The differences in the mean lesion size were assayed by Two-way ANOVA and Bonferroni post-hoc tests. 

*Parasite burden*

To determine the parasite burden, four mice were euthanized from each group at four weeks post-treatment, and their spleens were removed under aseptic conditions. 

The homogenized spleen tissue (10 mg) was cultured in 96-well cell plates containing RPMI-1640 medium with 100 IU/mL of penicillin, 10% FBS, and 100 µg/mL of streptomycin at serial dilutions of 1-10^-20^. Then, incubation was carried out at 26 °C. After seven and 14 days, to determine mobile promastigotes in each well, the plates were evaluated using an inverted microscope at 40X magnification. The final dilution with at least one motile promastigote was considered as the final titer. The following equation was used to determine the number of parasites:

parasite burden = -log (parasite dilution/tissue weight).

*Cytokine assay*

For IFN-γ and IL-4 measurements, splenocytes were exposed to soluble *Leishmania* antigen (SLA), obtained from stationary phase promastigotes according to previous studies (42). After euthanizing four mice from each group at four weeks post treatment, the spleens were extracted and aseptically homogenized in PBS. 

The supernatant was removed after double washing in PBS, and the cell pellets were resuspended in cold ammonium-chloride-potassium lysis buffer (5 mL; 0.15 M NH_4_Cl, 10 mM KHCO_3_, and 0.1 mM Na_2_EDTA); also, erythrocytes were lysed by incubating for 5 min at room temperature. After washing, 3 × 10^6^ cells/well were seeded in the plates, containing RPMI-1640 medium, which was supplemented with 100 μg/mL of streptomycin, 10% FBS, and 100 U/mL of penicillin; they were then exposed to 25 μg/mL of SLA at 37 °C. The supernatant was harvested after 72 h to assay cytokine levels (43). Also, ELISA kits (MABTECH, USA) were used, as outlined by the manufacturer, to measure IL-4 and IFN-γ. 

*Statistical analysis*

For data analysis, GraphPad Prism 5.0 (Graphpad Inc., USA) was used. The results were compared using parametric tests including unpaired samples *t*-test and One-way or Two-way ANOVA. *p*-value < 0.05 was considered significant. The values are presented as mean ± SD. Graphpad Prism was used for plotting the graphs. 

*Ethical considerations*

The Ethics Committee of Tarbiat Modares University approved all stages of the study, including animal maintenance and handling (ID.IR.TMU.REC.1394.259). 

## Results

*Proliferation of L. major promastigotes*

The number of *L. major* promastigotes was evaluated in the presence and absence of oxaliplatin and AmpB for 72 h at 24 °C. 

**Figure 1:**
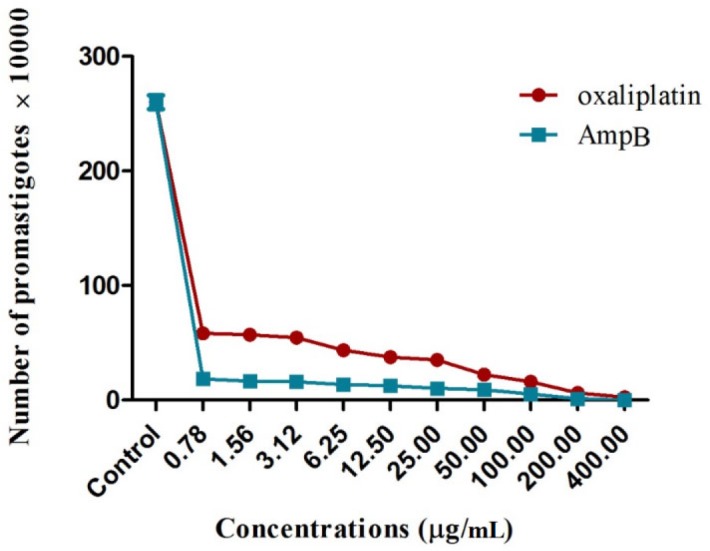
Antileishmanial effects of oxaliplatin or AmpB on the proliferations of *L. major *promastigotes after 72 h, *in-vitro*. All experiments were performed in triplicate

The results demonstrated that all concentrations of oxaliplatin significantly reduced the proliferation of *L. major *promastigotes, compared with those exposed to no drugs (*p* < 0.001) (Figure 1). The growth inhibitory effects of the drugs were dose–dependent. In other words, concentrations of 400 and 0.78 µg/mL resulted in the highest and lowest efficacies, respectively, on inhibiting the proliferation of *L. major* promastigotes. Moreover, it was shown that the exposure to 400 µg/mL of oxaliplatin inhibited the proliferation of *L. major *promastigotes completely after 72 h. IC_50_ values in the promastigotes treated with oxaliplatin and AmpB were 0.5 µg/mL and 0.28 µg/mL after 72 h, respectively.

*Metabolic activity of promastigotes and cytotoxicity*

Oxaliplatin efficacy on the metabolic activity of *L. major *promastigotes was measured by optical density (OD) following MTT assay to further confirm the microscopic examinations. Oxaliplatin reduced the metabolic activity of *L. major *promastigotes and showed similar activity of the reference drug AmpB, compared with those receiving no drug after 72 h (*p < *0.001). 

**Figure 2:**
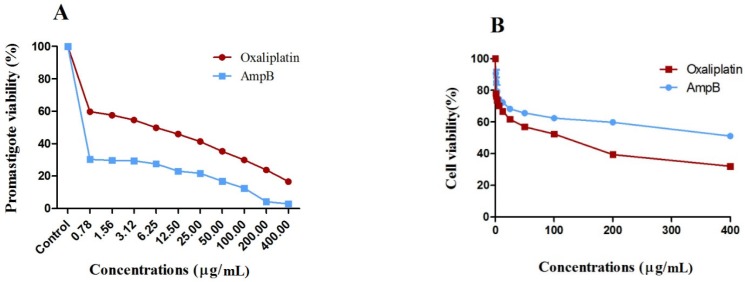
Percent viability of macrophages and *L. major *promastigotes exposed with oxaliplatin or AmpB after 72 h, *in-vitro*. (A) percent viability of *L. major *promastigotes and (B) percent viability of macrophage cells

The viability of *L. major* promastigotes showed the dose-dependent response (Figure 2A), and effectiveness enhanced with the increase in drug concentrations. Viability of *L. major* promastigotes was 59.2% and 15.7% for 0.78 and 400 µg/mL of oxaliplatin, respectively, while viability of promastigotes was 29.7% and 2% in the presence of 0.78 and 400 µg/mL of AmpB. 

Also, viability of macrophages treated with 0.78 and 400 µg/mL of oxaliplatin was 77.5% and 29.2%, and the corresponding values for macrophages exposed to AmpB were 89.8% and 49.4%, respectively after 72 h (Figure 2B). CC_50_ values in macrophages treated with oxaliplatin and AmpB were 66.78 and 184 µg/mL after 72 h respectively. Additionally, the results showed SI around 9.26 and 230 for oxaliplatin and AmpB after 72 h respectively (Table 1).

*Treatment of infected macrophages*

The activity of oxaliplatin and AmpB against *L. major* amastigotes was assayed, and the percentage of infectivity and IIR were calculated in the infected macrophages, treated with 6.25, 12.5, and 25 µg/mL of oxaliplatin or AmpB as the control. 

The results reported 89.3% of infection with *L. major* promastigotes in the stationary phase, and the mean number of amastigote/cell was 4.7 after 24 h. 

The results showed infectiveness reduction in macrophages treated with oxaliplatin and AmpB in comparison with untreated macrophages (*p < *0.001). The concentrations of these drugs revealed greater reduction in percentage of infectivity and IIR in macrophages so that percentage of infectivity was 4 and 2.33 in macrophages treated with 6.25 and 25 µg/mL of oxaliplatin, respectively 

(Table 2). 

The IC_50_ values of oxaliplatin and AmpB were 6.7 and 17 µg/mL against *L. major *amastiogtes, respectively. In addition, macrophages treated with 6.25, 12.5, and 25 µg/mL of oxaliplatin or AmpB showed a significant decrease in the percentage of infectivity and IIR, compared with the controls (*p <* 0.001), while no significant difference was found in infectivity or IIR of macrophages treated with oxaliplatin and AmpB.

*Apoptosis induced by oxaliplatin*

**Figure 3:**
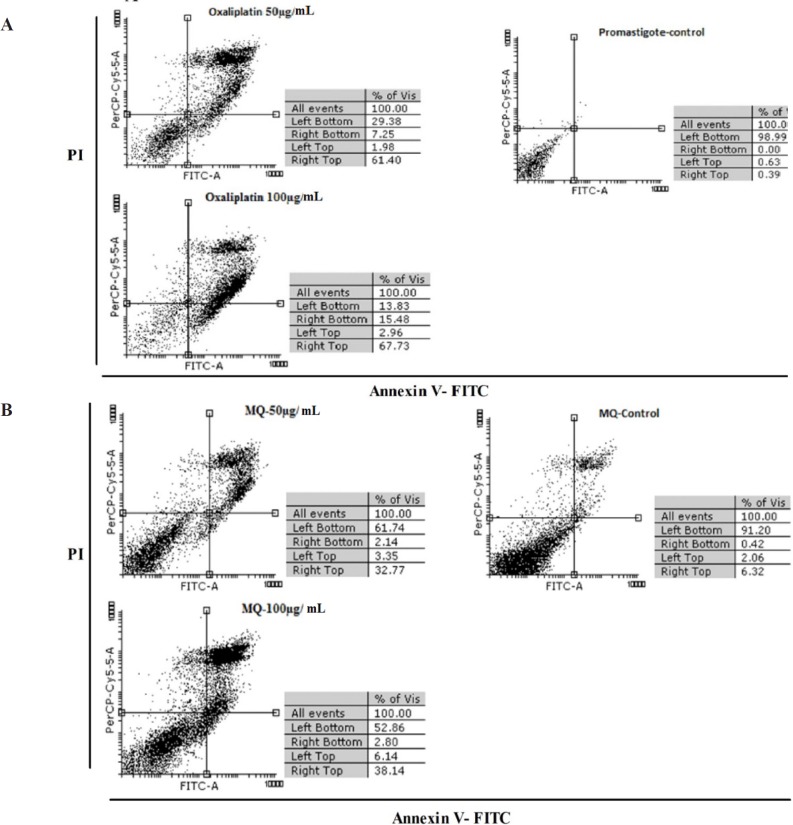
The results obtained from flow cytometry analysis on (A) *L. major *promastigotes and (B) Raw 264.7 macrophage Cells staining with Annexin V and Propidium Iodide after treatment with 50 and 100 µg/mL concentrations of oxaliplatin after 72 h, *in-vitro*

Figure 3 shows the results of flow cytometric analysis for promastigotes, infected macrophages, treated macrophages with 50 and 100 µg/mL of oxaliplatin, and macrophages treated with no drugs after 72 h. The results revealed necrosis and apoptosis in the promastigotes and macrophages. The rate of apoptosis caused by 50 and 100 μg/mL of oxaliplatin in the promastigotes was 68.65% and 83.21%, respectively, whereas in the control group (promastigotes) that received no drugs, 98.99% of the cells were alive and 0.39% were apoptotic. 

The percentage of apoptosis induced in macrophages exposed to 50 and 100 μg/mL concentrations of oxaliplatin was 34.91% and 40.94% respectively, while in the control group (macrophages) that was exposed to no drugs, 91.20% of the cells were alive and 6.74% were apoptotic (Figures 3A and 3B).

*Lesion size*

**Figure 4:**
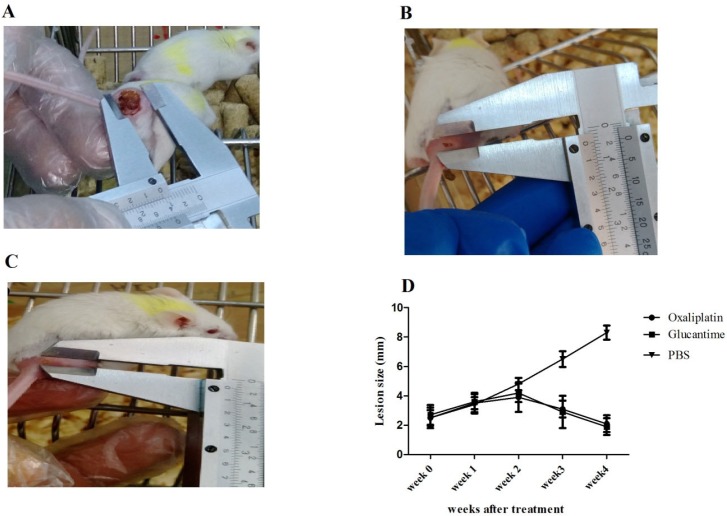
Lesion size in BALB/c mice infected with *L. major *promastigotes. (A-C) show the lesions in tail base of the mice that received PBS, glucantime and oxaliplatin respectively. (D) shows the mean ± SD of the mean lesion size which monitored weekly after treatment

Nodules developed in the injection site of parasites about 15-20 days post-infection. Lesion size in mice receiving PBS, glucantime, and oxaliplatin is shown in Figures 4A-4C.The results demonstrated that the mean lesion sizes reduced in groups receiving oxaliplatin and glucantime, compared with the PBS group in the second (*p < *0.01), third, and fourth week (*p < *0.0001) post-treatment. However, no difference in the mean lesion size was observed between the oxaliplatin and glucantime groups (Figure 4D).

*Parasite burden*

**Figure 5:**
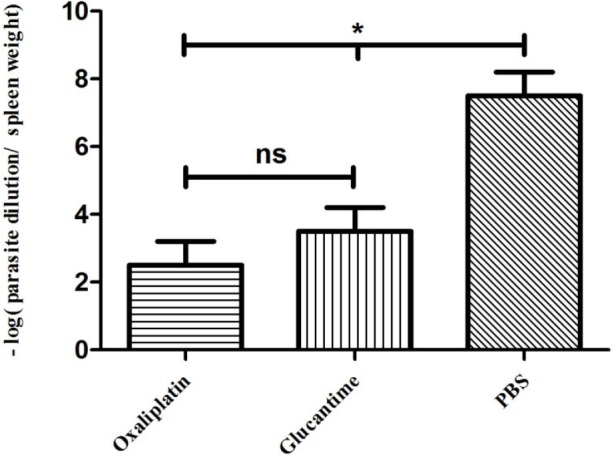
Parasite burden in the mice treated with oxaliplatin, glucantime or PBS. The number of promastigotes was compared between all groups by One-way ANOVA test. The asterisk sign shows the significant difference between the number of promastigotes as determined (*p *< 0.05 represented as * and ns. represented as non-significant). Each bar shows mean ± SD of the number of promastigotes obtained from each group

The parasite load decreased significantly in groups treated with oxaliplatin and glucantime compared with the groups receiving PBS (*p < *0.05), whereas no significant difference was reported in parasite burden between mice treated with oxaliplatin and those treated with glucantime (Figure 5).

*Cytokine assay*

**Figure 6:**
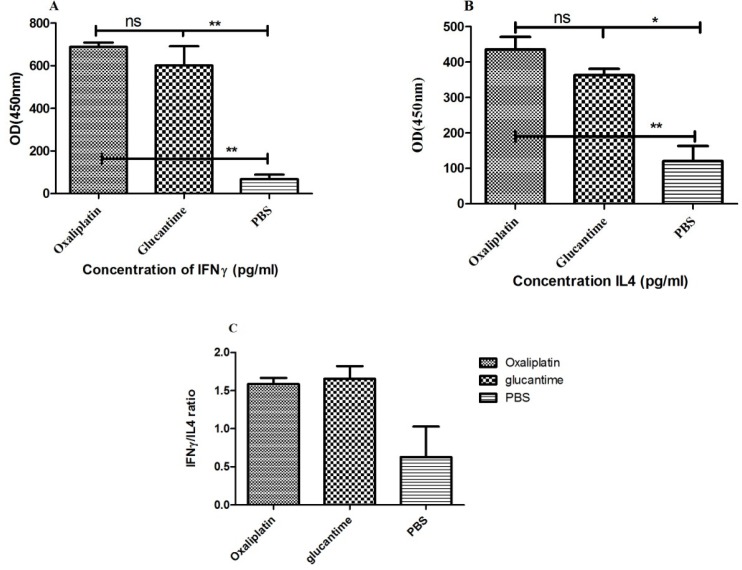
Cytokine levels were produced by the splenocytes obtained from BALB/c mice received oxaliplatin, glucantime or PBS. Cytokine levels were compared with One-way ANOVA. Each bar represents Mean ± SD of cytokine concentrations. (A and B) indicate respectively IFN-γ and IL-4 levels and (C) shows IFN-γ/IL-4 ratio. The asterisk sign shows the significant difference between cytokine values as determined (*p *< 0.05 represented as *, *p *< 0.01 represented as ** and ns. represented as non-significant)

The IFN-γ and IL-4 levels were significantly different between the PBS group and mice treated with oxaliplatin and glucantime (Figures 6A and 6B). IFN-γ level increased in mice that received oxaliplatin or glucantime versus the PBS group (*p <* 0.01). 

Nevertheless, a significant increase was found in the IL-4 level of the mice treated with oxaliplatin in comparison with the PBS group (*p < *0.05), similar to the glucantime group (*p < *0.01). 

However, IFN-γ and IL-4 levels were not significantly different between the oxaliplatin and glucantime groups. The IFN-γ/IL-4 ratio in mice treated with oxaliplatin, glucantime, and PBS was 1.58, 1.65, and 0.63, respectively.

**Table 1:** Determination of IC50, CC50 and SI of promastigotes and Raw 264.7 macrophage cells treated with oxaliplatin and AmpB*, in-vitro *(µg/mL)

**SI** ^c^	**CC**_50_^b^	**IC**_50_^a^	**Drug**
9.26	66.7 ± 1.82	7.19 ± 0.85	Oxaliplatin
230	184 ± 2.26	0.8 ± 0.65	AmpB

^a^50% inhibitory concentration of promastigote growth.

^b^50% inhibitory concentration of the macrophages.

^c^selectivity index was measured by calculation of CC_50_/IC_50_ ratio.

The data were expressed as mean ± standard errors. IC_50_ and CC_50_ was calculated by non-linear regression analysis.

**Table 2:** Determination of infection index rates and infectivity% of macrophages infected by *L. major* promastigotes and exposed to different concentrations of oxaliplatin and AmpB. The results were expressed as mean ± standard deviation of Infectivity% and IIR in untreated and treated macrophages, *in-vitro* (µg/mL)

**AmpB/IC**_50_ ** (17 ± 1.23)**		**Oxaliplatin/IC**_50_ ** (6.7 ± 0.82)**		**Concentrations**
**IIR**	**Infectivity (%)**	**IIR**	**Infectivity (%)**	
4.7 ± 0.2	89.3 ± 0.03	4.7 ± 0.2	89.3 ± 0.03	Negative control
2.57 ± 0.04	10 ± 2.64	2.28 ± 0.02	4 ± 1.52	6.25
2.47 ± 0.07	8.3 ± 1.52	2.33 ± 0.03	3 ± 1	12.5
2.37 ± 0.07	6.33 ± 1.15	2.19 ± 0.02	2.33 ± 0.57	25

## Discussion

Current treatments against leishmaniasis are limited because of unacceptable toxicity, high costs, and appearance of drug resistance in some endemic areas (44). Therefore, researchers are constantly seeking new drugs with potential leishmanicidal activities and low toxicities. In previous studies, the anti-leishmanial activity of some anti-cancer drugs such as miltefosine (45), tamoxifen (46), artemisinin (47), and 5-fluorouracil (30) has been reported. The anti-leishmanial effects of miltefosine are caused by apoptosis in cell membrane phospholipids and formation of inflammatory cytokines (*e.g.*, TNF-α and IL-12) (45).

Doxorubicin and Doxil are two other anticancer drugs that have shown leishmanicidal efficacies by reducing growth in both* L. major* promastigotes and amastigotes (48). Platinum compounds including cisplatin and oxaliplatin are standard drugs in cancer treatment and have the same effect mechanism at DNA as their pharmacological target. However, their toxicities and specificity of tumor tissues are different (49). The antileishmanial effects of cisplatin alone or in combination with herbal compounds against *Leishmania donovani* were confirmed *in-vitro* and *in-vivo*; however, application of this drug was limited because of its toxic effects on tissues (50).

Oxaliplatin is generally recognized as a third-generation antineoplastic drug, used either alone or in combination with other drugs (51). Oxaliplatin is used as first or second-line treatment for advanced colorectal cancer, and it has shown more safety with less nephrotoxicity or ototoxicity than other platinum derivatives (52). Oxaliplatin has also shown therapeutic efficacies against ovarian cancer with better tolerability and equal effectiveness compared with carboplatin (53). Oxaliplatin, by formation of DNA lesions, DNA synthesis arrest, inhibition of RNA synthesis, and stimulation of immunologic reactions, leads to apoptosis in cancer cells (54).

Generally, oxaliplatin can induce both apoptosis and necrosis (55). The anticancer effects of oxaliplatin improve when it is associated with other cytotoxic drugs, such as 5-fluorouracil, leucovorin, or Vorinostat (51, 56). These results encourage researchers to examine the anti-leishmanial activities of oxaliplatin against *L. major* both *in-vitro* and *in-vivo*.

The current findings demonstrated that oxaliplatin inhibited *L. major* promastigote proliferation in time and concentration-dependent manners, similar to the reference drug (AmpB); its effectiveness improved with an increase in drug concentration and duration. Also, 400 µg/mL of oxaliplatin was the most effective in inhibiting promastigote growth after 72 h of incubation. In addition, the results proved that viability of *L. major *promastigotes and macrophages exposed to oxaliplatin reduced in a concentration-dependent manner, same as the reference drug, AmpB. The viability of *L. major* promastigotes was 59.2% and 15.7% for 0.78 and 400 µg/mL of oxaliplatin, respectively, and viability of macrophages treated with 0.78 and 400 µg/mL of oxaliplatin was 77.5% and 29.2%. 

Although the present results showed the antileishmanial activity of oxaliplatin on *L. major* promastigotes *in-vitro*, it also had cytotoxic effects on Raw 264.7 macrophage cells. Fazio *et al*. reported that oxaliplatin induces suicidal death in human erythrocytes through cell shrinkage and membrane scrambling by phosphatidylserine translocation to the erythrocyte surface (55). Therefore, safety of oxaliplatin for human cells is questionable. Consequently, oxaliplatin reduced the metabolic activity of *L. major* promastigotes just like AmpB. Oxaliplatin also inhibited the growth of amastigote forms inside macrophages. Percentage of live amastigotes in infected macrophages reduced in the presence of oxaliplatin or AmpB so that percentage of infectivity and IIR decreased in the macrophages exposed with oxaliplatin and AmpB compared with those in control group. 

Furthermore, 25 µg/mL of oxaliplatin caused a 97% reduction in the percentage of infectivity of macrophages, while AmpB reduced it about 93%. Therefore, the findings showed that oxaliplatin may act as a suitable antileishmanial agent and may even be more effective than AmpB against *L. major* amastigotes. 

These results confirmed the findings reported by Shokri *et al. *who found that the antileishmanial effects of doxorubicin and Doxil at higher concentrations were greater than meglumine antimoniate (48). Also, tamoxifen and ZnO nanoparticles inhibited the proliferation of promastigote and amastigote stages of *L. major *in a concentration and time dependent manner (57).

Oxaliplatin concentrations of 50 and 100 μg/mL induced 68.65% and 83.21% apoptosis in *L. major* promastigotes, respectively, and its apoptotic effects increased at higher drug concentrations with slight alterations in necrotic values. Ghaffarifar *et al.* (58) showed 49.03% and 81.31% apoptosis in *L. major* promastigotes at the same concentrations of artemisinin, respectively. Similar results have been obtained with other anticancer drugs; for instance, miltefosine and tamoxifen revealed their leishmanicidal effectiveness by inducing apoptosis in the promastigotes of *Leishmania* species (59).

The results of the murine model in this study showed that the mice treated with glucantime and oxaliplatin developed smaller lesions, compared with the control group, while the oxaliplatin and glucantime groups were not significantly different. However, none of those could heal the lesions completely. Also, the parasite burden decreased in the oxaliplatin and glucantime groups. In the current study, a relationship between lesion size and load parasite was observed. These findings are in line with the analysis of antileishmanial activity of cisplatin, which reduced parasite burden in VL-infected mice caused by *L. donovani* (35).

In another study, hydrogels containing the active extract of *Libidibia ferrea *led to a reduction in parasite burden and lesion size in *L. amazonensis-*infected golden hamsters (60). Furthermore, similar to glucantime, the splenocytes of mice treated with oxaliplatin produced higher IFN-γ levels, compared with the PBS group. Although IL-4 levels also increased in groups receiving glucantime or oxaliplatin, the IFN-γ/IL-4 ratio in the glucantime and oxaliplatin groups was 2.5 and 2.61 times higher than that of the PBS group, respectively. Oxaliplatin may reduce disease caused by *L. major* in BALB/c mice by increasing the inflammatory cytokines.

## Conclusion

The current results imply an effective antileishmanial activity for oxaliplatin against *L. major* promastigotes and intracellular amastigotes. Additionally, use of oxaliplatin was promising in the treatment of *L. major* infected BALB/c mice by decreasing the parasite burden and lesion size and by increasing IFN-γ production. These findings suggest that oxaliplatin can be used as a drug either alone or along with other antileishmanial products for CL treatment. Despite the anti-leishmanial activity of oxaliplatin on *L. major* promastigotes *in-vitro*, the cytotoxic effects on macrophage cells were reported. Therefore, the safety of oxaliplatin for human cells is questionable, and there may be concerns about the use of this drug. The present research suggests more investigations on the anti-leishmanial effects of oxaliplatin on other species of the genus *Leishmania* and its toxicity on different cell lines.

## References

[1] Desjeux P (2004). Leishmaniasis: current situation and new perspectives. Comp. Immunol. Microb.

[2] Alvar J, Velez ID, Bern C, Herrero M, Desjeux P, Cano J, Jannin J (2012). Leishmaniasis worldwide and global estimates of its incidence. PLoS One.

[3] Tafaghodi M, Eskandari M, Khamesipour A, Jaafari MR (2016). Immunization against cutaneous leishmaniasis by alginate microspheres loaded with autoclaved Leishmania major (alm) and quillaja saponins. Iran. J. Pharm. Res..

[4] Abamor ES, Allahverdiyev AM (2016). A nanotechnology based new approach for chemotherapy of Cutaneous Leishmaniasis: TIO2@ AG nanoparticles–Nigella sativa oil combinations. Exp. Parasitol.

[5] Kumar R, Bumb RA, Ansari NA, Mehta RD, Salotra P (2007). Cutaneous leishmaniasis caused by Leishmania tropica in Bikaner, India: parasite identification and characterization using molecular and immunologic tools. Am. J. Trop. Med. Hyg.

[6] Malmasi A, Ardestani BZ, Mohebali M, Akhoundi B, Ziaie S, Masoudifard M, Khorshid HK (2014). Evaluation of a novel herbal immunomodulator drug (IMOD) in treatment of experimental canine visceral leishmaniasis. Iran. J. Pharm. Res.

[7] Haddad MHF, Ghasemi E, Maraghi S, Tavala M (2016). Identification of Leishmania species isolated from human cutaneous leishmaniasis in Mehran, Western Iran using nested PCR. Iran. J. Parasitol.

[8] Abdoli A, Maspi N, Ghaffarifar F (2017). Wound healing in cutaneous leishmaniasis: a double edged sword of IL-10 and TGF-β. Comp. Immunol. Microb.

[9] Mansour R, Haouas N, Kahla-Nakbi AB, Hammami S, Mighri Z, Mhenni F, Babba H (2013). The effect of Vitis vinifera L leaves extract on Leishmania infantum. Iran. J. Pharm. Res.

[10] Saroufim M, Charafeddine K, Issa G, Khalifeh H, Habib RH, Berry A, Ghosn N (2014). Ongoing epidemic of cutaneous leishmaniasis among Syrian refugees, Lebanon. Emerg. Infect. Dis.

[11] González C, Wang O, Strutz SE, González-Salazar C, Sánchez-Cordero V, Sarkar S (2010). Climate change and risk of leishmaniasis in North America: predictions from ecological niche models of vector and reservoir species. PLOS Negl. Trop. Dis.

[12] Salam N, Al-Shaqha WM, Azzi A (2014). Leishmaniasis in the Middle East: incidence and epidemiology. PLOS Negl. Trop. Dis.

[13] de Menezes JPB, Guedes CES, Petersen ALdOA, Fraga DBM, Veras PST (2015). Advances in development of new treatment for leishmaniasis. Biomed. Res. Int.

[14] Ebrahimisadr P, Ghaffarifar F, Horton J, Dalimi A, Sharifi Z (2018). Apoptotic effect of morphine, imiquimod and nalmefene on promastigote, infected and uninfected macrophages with amastigote of Leishmania major by flow cytometry. Iran. J. Pharm. Res.

[15] Sifaoui I, López-Arencibia A, Martín-Navarro CM, Reyes-Batlle M, Mejri M, Valladares B, Lorenzo-Morales J (2017). Selective activity of oleanolic and maslinic acids on the amastigote form of Leishmania Spp. Iran. J. Pharm. Res.

[16] Kato KC, Morais-Teixeira E, Reis PG, Silva-Barcellos NM, Salaün P, Campos PP, Corrêa-Junior JD (2014). Hepatotoxicity of pentavalent antimonial drug: possible role of residual Sb (III) and protective effect of ascorbic acid. Antimicrob. Agents Chemother.

[17] Oliveira LF, Schubach AO, Martins MM, Passos SL, Oliveira RV, Marzochi MC, Andrade CA (2011). Systematic review of the adverse effects of cutaneous leishmaniasis treatment in the new world. Acta Trop.

[18] Arana B, Rizzo N, Diaz A (2001). Chemotherapy of cutaneous leishmaniasis: a review. Med. Microbiol. Immun.

[19] Singh N, Kumar M, Singh RK (2012). Leishmaniasis: current status of available drugs and new potential drug targets. Asian Pac. J. Trop. Med.

[20] Shahidi Dadras M, Mirzaei A, Kazemi B, Nabai L, Sharifian A (2010). A comparative study of aminosidine sulfate, meglomine antimoniate, combination of both and glucantime in murine leishmaniasis treatment of cutaneous leishmaniasis, caused by Leishmania tropica, with topical application of paromomycin 20% in BALB-c mice. Iran. J. Pharm. Res.

[21] Wijnant GJ, Van Bocxlaer K, Yardley V, Murdan S, Croft SL (2017). Efficacy of paromomycin-chloroquine combination therapy in experimental cutaneous leishmaniasis. Antimicrob. Agents Chemother.

[22] Croft SL, Sundar S, Fairlamb AH (2006). Drug resistance in leishmaniasis. Clin. Microbiol. Rev.

[23] Abazari R, Mahjoub AR, Molaie S, Ghaffarifar F, Ghasemi E, Slawin AM, Carpenter-Warren CL (2018). The effect of different parameters under ultrasound irradiation for synthesis of new nanostructured Fe3O4@ bio-MOF as an efficient anti-leishmanial in-vitro and in-vivo conditions. Ultrason. Sonochem.

[24] Soudi S, Hashemi SM, Zavaran Hosseini A, Ghaemi A, Asghari Jafarabadi M (2010). Antileishmanial effect of Echinacea purpurea root extract cultivated in Iran. Iran. J. Pharm. Res.

[25] Oliveira G (2014). Cancer and parasitic infections: similarities and opportunities for the development of new control tools. Rev. Soc. Bras. Med. Tro.

[26] Eastman RT, Fidock DA (2009). Artemisinin-based combination therapies: a vital tool in efforts to eliminate malaria. Nat. Rev. Microbiol.

[27] Douglas NM, Anstey NM, Angus BJ, Nosten F, Price RN (2010). Artemisinin combination therapy for vivax malaria. Lancet Infect. Dis.

[28] Olliaro P, Taylor W (2004). Developing artemisinin based drug combinations for the treatment of drug resistant falciparum malaria: A review. J. Postgrad. Med.

[29] Beckmann S, Grevelding C (2010). Imatinib has a fatal impact on morphology, pairing stability and survival of adult Schistosoma mansoni in-vitro. Int. J. Parasitol.

[30] Dalimi A, Ghaffarifar F, Sadraei J (2014). Cytotoxic effects of 5-fluorouracil on Leishmania major promastigotes and induction of apoptosis in the parasite. J. Ilam Uni. Med. Sci.

[31] Trinconi CT, Reimão JQ, Coelho AC, Uliana SR (2016). Efficacy of tamoxifen and miltefosine combined therapy for cutaneous leishmaniasis in the murine model of infection with Leishmania amazonensis. J. Antimicrob. Chemother.

[32] Kaur T, Makkar P, Randhawa K, Kaur S (2013). Antineoplastic drug, carboplatin, protects mice against visceral leishmaniasis. Parasitol. Res.

[33] Croft SL, Engel J (2006). Miltefosine—discovery of the antileishmanial activity of phospholipid derivatives. Trans. Royal Soc. Trop. Med. Hyg.

[34] Mani S, Graham MA, Bregman DB, Ivy P, Chaney SG (2002). Oxaliplatin: a review of evolving concepts. Cancer Invest.

[35] Kaur S, Sachdeva H, Dhuria S, Sharma M, Kaur T (2010). Antileishmanial effect of cisplatin against murine visceral leishmaniasis. Parasitol. Int.

[36] Ebrahimisadr P, Ghaffarifar F, Hassan ZM (2013). In-vitro evaluation of antileishmanial activity and toxicity of artemether with focus on its apoptotic effect. Iran. J. Pharm. Res.

[37] Sundar S, Chakravarty J (2010). Antimony toxicity. Int. J. Environ. Res. Public Health.

[38] da Luz JS, de Oliveira EB, Martins MC, Silva NHd, Alves LC, dos Santos FA, da Silva LL (2015). Ultrastructural analysis of leishmania infantum chagasi Promastigotes forms treated in-vitro with usnic acid. Sci. World J.

[39] Shokri A, Sharifi I, Khamesipour A, Nakhaee N, Harandi MF, Nosratabadi J, Parizi MH (2012). The effect of verapamil on in-vitro susceptibility of promastigote and amastigote stages of Leishmania tropica to meglumine antimoniate. Parasitol. Res.

[40] Duarte MC, dos Reis Lage LM, Lage DP, Mesquita JT, Salles BCS, Lavorato SN, Menezes-Souza D (2016). An effective in-vitro and in-vivo antileishmanial activity and mechanism of action of 8-hydroxyquinoline against Leishmania species causing visceral and tegumentary leishmaniasis. Vet. Parasitol.

[41] Abamor ES (2017). Antileishmanial activities of caffeic acid phenethyl ester loaded PLGA nanoparticles against Leishmania infantum promastigotes and amastigotes in-vitro. Asian Pac. J. Trop. Med.

[42] Maspi N, Ghaffarifar F, Sharifi Z, Dalimi A, Dayer MS (2017). Immunogenicity and efficacy of a bivalent DNA vaccine containing LeIF and TSA genes against murine cutaneous leishmaniasis. Apmis.

[43] Maspi N, Ghaffarifar F, Sharifi Z, Dalimi A, Dayer MS (2018). Comparative assessment of induced immune responses following intramuscular immunization with fusion and cocktail of LeIF, LACK and TSA genes against cutaneous leishmaniasis in BALB/c mice. Arch. Immunol. Ther. Exp..

[44] Chávez-Fumagalli MA, Ribeiro TG, Castilho RO, Fernandes SOA, Cardoso VN, Coelho CSP, Mendonça DVC (2015). New delivery systems for amphotericin B applied to the improvement of leishmaniasis treatment. Rev. Soc. Bras. Med. Tro..

[45] Paris C, Loiseau PM, Bories C, Bréard J (2004). Miltefosine induces apoptosis-like death in Leishmania donovani promastigotes. Antimicrob. Agents Chemother.

[46] Miguel DC, Yokoyama-Yasunaka JK, Uliana SR (2008). Tamoxifen is effective in the treatment of Leishmania amazonensis infections in mice. PLOS Negl. Trop. Dis.

[47] Cortes S, Albuquerque A, Cabral LI, Lopes L, Campino L, Cristiano ML (2015). In-vitro susceptibility of Leishmania infantum to Artemisinin derivatives and selected trioxolanes. Antimicrob. Agents Chemother.

[48] Shokri A, Akhtari J, Keighobadi M, Fakhar M, Teshnizi SH, Emami S, Sadjjadian S (2017). Promising antileishmanial effectiveness of doxorubicin and Doxil against Leishmania major: an in-vitro assay. Asian Pac. J. Trop. Med.

[49] Becker JP, Weiss J, Theile D (2014). Cisplatin, oxaliplatin, and carboplatin unequally inhibit in-vitro mRNA translation. Toxicol. Lett.

[50] Sachdeva H, Kaur S (2018). Cisplatin along with herbal drug treatment reduces the percentage of regulatory T cells and decreased the severity of experimental visceral leishmaniasis. J. Microbiol. Immunol.

[51] Falcone A, Ricci S, Brunetti I, Pfanner E, Allegrini G, Barbara C, Crino L (2007). Phase III trial of infusional fluorouracil, leucovorin, oxaliplatin, and irinotecan (FOLFOXIRI) compared with infusional fluorouracil, leucovorin, and irinotecan (FOLFIRI) as first-line treatment for metastatic colorectal cancer: the Gruppo Oncologico Nord Ovest. J. Clin. Oncol.

[52] Montagnani F, Turrisi G, Marinozzi C, Aliberti C, Fiorentini G (2011). Effectiveness and safety of oxaliplatin compared to cisplatin for advanced, unresectable gastric cancer: a systematic review and meta-analysis. Gastric Cancer.

[53] Bogliolo S, Cassani C, Gardella B, Musacchi V, Babilonti L, Venturini PL, Ferrero S, Spinillo A (2015). Oxaliplatin for the treatment of ovarian cancer. Expert Opin. Inv. Drugs.

[54] Alcindor T, Beauger N (2011). Oxaliplatin: a review in the era of molecularly targeted therapy. Curr. Oncol..

[55] Fazio A, Briglia M, Faggio C, Alzoubi K, Lang F (2015). Oxaliplatin induced suicidal death of human erythrocytes. Cell. Physiol. Biochem.

[56] Liao B, Zhang Y, Sun Q, Jiang P (2018). Vorinostat enhances the anticancer effect of oxaliplatin on hepatocellular carcinoma cells. Cancer Med.

[57] Doroodgar M, Delavari M, Doroodgar M, Abbasi A, Taherian AA, Doroodgar A (2016). Tamoxifen induces apoptosis of leishmania major promastigotes in-vitro. Korean J. Parasitol.

[58] Ghaffarifar F, Heydari FE, Dalimi A, Hassan ZM, Delavari M, Mikaeiloo H (2015). Evaluation of apoptotic and antileishmanial activities of Artemisinin on promastigotes and BALB/C mice infected with Leishmania major. Iran. J. Parasitol.

[59] Sen R, Bandyopadhyay S, Dutta A, Mandal G, Ganguly S, Saha P, Chatterjee M (2007). Artemisinin triggers induction of cell-cycle arrest and apoptosis in Leishmania donovani promastigotes. J. Med. Microbiol.

[60] Comandolli-Wyrepkowski CD, Jensen BB, Grafova I, Santos PAD, Barros AMC, Soares FV, Barcellos JFM (2017). Antileishmanial activity of extracts from Libidibia ferrea: development of in-vitro and in-vivo tests. Acta Amazonica.

